# Interspecies transmission to bovinized transgenic mice uncovers new features of a CH1641-like scrapie isolate

**DOI:** 10.1186/s13567-018-0611-1

**Published:** 2018-11-28

**Authors:** Kohtaro Miyazawa, Kentaro Masujin, Yuichi Matsuura, Yoshifumi Iwamaru, Takashi Yokoyama, Hiroyuki Okada

**Affiliations:** 1 Prion Disease Unit, Division of Transboundary Animal Disease, National Institute of Animal Health (NIAH), National Agriculture and Food Research Organization (NARO), Tsukuba, Ibaraki Japan; 2 Exotic Disease Research Unit, Division of Transboundary Animal Diseases, NIAH, NARO, Kodaira, Tokyo Japan; 3 Department of Planning and General Administration, NIAH, NARO, Tsukuba, Ibaraki Japan

## Abstract

In animal prion diseases, including bovine spongiform encephalopathy (BSE) in cattle, chronic wasting disease in cervids, and scrapie in sheep and goats, a disease-associated isoform of prion protein (PrP^d^) accumulates in the brains of affected animals. Although the CH1641 scrapie isolate was experimentally established in the UK, a few natural CH1641-like scrapie cases have been reported in France and the UK. The molecular mass of the unglycosylated protease-resistant core of PrP^d^ (PrPres) is known to be similar between CH1641-like scrapie and experimental BSE in sheep. We previously established an experimental CH1641-like scrapie isolate (Sh294) from a natural classical scrapie case. Here, we demonstrated that the Sh294 isolate was independent of both classical and atypical BSEs by cross-species transmission to bovine PrP overexpressing (TgBoPrP) mice and wild-type mice. Interestingly, we found that the Sh294 isolate altered its host range by the transmission to TgBoPrP mice, and we succeeded in the first stable reproduction of CH1641-like scrapie specific PrPres banding patterns with the ~12-kDa small C-terminal fragment in wild-type mice. This study provides new insight into the relationship between CH1641-like scrapie isolates and BSEs. In addition, interspecies transmission models such as we have demonstrated here could be a great help to investigate the origin and host range of animal prions.

**Electronic supplementary material:**

The online version of this article (10.1186/s13567-018-0611-1) contains supplementary material, which is available to authorized users.

## Introduction

Transmissible spongiform encephalopathies (TSEs), or prion diseases, are fatal neurodegenerative disorders that affect both humans and animals [1]. Prion diseases are characterized by spongiform changes and accumulation of a disease-associated isoform of prion protein (PrP^d^), which is generated by post-transcriptional modification of host cellular prion protein (PrP^C^), in the brains of affected hosts [2]. PrP^d^ (or prion) is believed to be a causative agent of TSEs [3]. Bovine spongiform encephalopathy (BSE) is a prion disease in cattle [4] that was first identified in the UK and spread to European as well as North American countries and Japan through feeding of BSE-contaminated meat and bone meal [5]. This epidemic BSE is now called classical (C-) BSE because two other atypical disease phenotypes (H-BSE and L-BSE) have been reported. Based on the molecular weight of the proteinase K-resistant core of PrP^d^ (PrPres) determined by Western blot (WB) analysis, H-BSE exhibits higher-molecular-weight PrPres banding patterns than C-BSE does [6], whereas L-BSE shows a lower-molecular-weight banding pattern [7]. Despite extensive studies, the origin of BSEs remains unknown.

Scrapie is a prion disease in sheep and goats that has been suspected to be the origin of BSEs since the discovery of the first C-BSE case in 1986. Scrapie is also classified into two disease phenotypes, termed classical and atypical scrapie [8]. To test the scrapie origin hypothesis, several experimental transmissions of classical scrapie isolates to cattle have been performed in the USA and UK [9–13]. However, the biochemical and pathological properties of accumulated PrP^d^ in the brains of cattle infected with classical scrapie isolates are not consistent with those of cattle infected with C-BSE.

The CH1641 scrapie isolate was experimentally established from a case of natural classical scrapie that occurred in the UK [14]. In contrast to classical scrapie isolates, this isolate is biochemically characterized by a lower molecular mass of the unglycosylated PrPres compared with that of classical scrapie isolates and an additional ~12-kDa small C-terminal PrPres fragment [15, 16]. To date, a few natural CH1641-like scrapie cases have been reported in France and the UK [17, 18]. However, no transmission studies of CH1641 or CH1641-like scrapie isolates to cattle have been reported. In our previous work, we experimentally established a CH1641-like scrapie isolate (Sh294) from a case of natural classical scrapie that occurred in the USA [19]. Sh294 cannot transmit to wild-type mice, similar to the British CH1641 scrapie isolate, but can transmit to bovine PrP-overexpressing (TgBoPrP) mice [14, 19]. Interestingly, these mice showed clinical signs of the disease similar to those of L-BSE infection.

In this study, we aimed to determine the relationship between CH1641-like scrapie and BSEs (particularly L-BSE) in cattle by further investigating the biological and biochemical properties of the Sh294 isolate in TgBoPrP mice. Because we have reported that L-BSE acquired transmissibility to wild-type mice after passage in sheep [20], we next attempted to transmit the Sh294 isolate passaged in TgBoPrP mice (designated as TgBo-passaged Sh294) to wild-type mice to examine the effects of interspecies transmission on the host range of the CH1641-like scrapie isolate.

## Materials and methods

### Mouse inoculation

TgBoPrP mice were intracerebrally inoculated with 20 µL of 10% (weight/volume) brain stem homogenates from a Corriedale sheep with experimentally developed CH1641-like scrapie (Sh294) [19]. To compare the biological and biochemical properties of this CH1641-like scrapie isolate with those of three different BSE strains, TgBoPrP mice were also intracerebrally inoculated with 20 µL of 10% brain stem homogenates from cattle experimentally affected with L-, C-, and H-BSE. PrPres-positive mouse brains were used for subsequent passages in TgBoPrP mice. TgOvPrP59 mice, which overexpress ovine PrP_ARQ_ (A136, R154, Q171) under the control of the neuron-specific enolase promoter [21] (a kind gift from Dr. Thierry Baron, Agence Nationale de Sécurité Sanitaire, Lyon, France) were also used for the intracerebral inoculation of the Sh294 isolate. To test the cross-species transmission ability of the TgBo-passaged Sh294 isolate, further transmission studies to wild-type mice were conducted. Three-week-old outbred ICR mice (SLC, Shizuoka, Japan) were intracerebrally inoculated with 20 µL of 10% brain homogenates from TgBoPrP mice infected with Sh294. To confirm the adaptation of the TgBo-passaged Sh294 isolate in ICR mice, subsequent passages were conducted. Additionally, subsequent passages of sheep-passaged L-BSE [22] in ICR mice were also conducted to compare with TgBo-passaged Sh294 in ICR mice. Mouse-adapted C-BSE (mBSE) and three laboratory scrapie strains (ME7, 22L, and Chandler) passaged in ICR mice were also used in this study. Mice were monitored daily for the presence of clinical signs, such as rough fur, hunched posture, and emaciation. All challenged mice were sacrificed under sevoflurane anesthesia when they began to exhibit signs of distress or progression of the disease was evident. The survival periods in this study were determined as the time elapsed from inoculation to the clinical endpoint or sudden death of mice that were pathologically and/or biochemically positive for PrP^d^.

### Antibodies used in this study

Information on monoclonal anti-PrP antibodies used in this study is detailed in Table 1. Polyclonal rabbit anti-glial fibrillary acidic protein (GFAP) antibodies (Dako, Carpinteria, CA, USA) were also used for the immunostaining of astrocytes.

**Table 1:** **Monoclonal anti-PrP antibodies used in this study**

Clone	Epitope location on bovine PrP	Species reactivity	Vendor	Dilution
P4	101–107	Cattle, sheep	R-Biopharm, Darmstadt, Germany	1: 5000 (WB)
6H4	156–163	Cattle, sheep, mice	Prionics, Schlieren, Switzerland	1: 5000 (WB)
SAF84	175–180	Cattle, sheep, mice	Bertin Pharma, Montigny le Bretonneux, France	1: 5000 (WB)
31C6	155–163	Cattle, sheep, mice	Frontier Science, Hokkaido, Japan	1: 1800 (IHC)
T1	149–155	Cattle, sheep, mice	Not for sale (See reference number 41.)	1: 4500 (IHC)

### Histopathology and immunohistochemistry

The left hemispheres of mouse brains were fixed in 10% buffered formalin containing 10% methanol. Formalin-fixed brains were immersed in 98% formic acid for 60 min to reduce infectivity, embedded in paraffin, and sectioned for histological evaluation by staining with hematoxylin and eosin (H&E). For PrP^d^ immunohistochemistry, 31C6 [23] and T1 [24] monoclonal antibodies (mAbs) were used. After appropriate epitope retrieval with hydrate autoclaving, brain sections were incubated with primary antibodies. After washing, the sections were incubated with an anti-mouse, universal horseradish peroxidase (HRP)-conjugated polymer (Nichirei Histofine Simple Stain MAX-PO (M); Nichirei Biosciences Inc., Tokyo, Japan), and PrP^d^ was visualized with 3,3′-diaminobenzidine tetrachloride as the chromogen. Finally, the sections were lightly counterstained with Mayer’s hematoxylin. For dual immunofluorescence, the sections were incubated for 60 min with T1 mAbs and polyclonal rabbit anti-GFAP antibodies. After washing, the sections were incubated with Alexa Fluor 488-conjugated goat anti-mouse IgG (1:450; Molecular Probes, Portland, OR, USA) for PrP^d^ and Alexa Fluor 546-conjugated goat anti-rabbit IgG (1:450; Molecular Probes) for GFAP. Finally, the sections were counterstained with Topro-3 (1:2000; Fisher Scientific K.K., Yokohama, Japan). Immunofluorescence was evaluated using a Zeiss LSM 510 laser scanning confocal microscope (Carl Zeiss, Oberkochen, Germany).

### PrPres detection by WB

Brain homogenates (20%) were mixed with an equal volume of detergent buffer containing 4% (w/v) Zwittergent 3–14 (Merck Japan, Tokyo, Japan), 1% (w/v) Sarkosyl (Sigma-Aldrich Japan, Tokyo, Japan), 100 mM NaCl, and 50 mM Tris–HCl (pH 7.6), and incubated for 30 min with collagenase (Wako, Osaka, Japan; final concentration of 500 µg/mL) at 37 °C. Samples prepared from brains were then incubated for 30 min with proteinase K (PK; Roche Diagnosis Japan, Tokyo, Japan; final concentration of 40 µg/mL) at 37 °C. PK digestion was terminated with 2 mM 4-(2-aminoethyl) benzenesulfonyl fluoride hydrochloride (Pefabloc; Roche Diagnostics Japan). Samples were mixed with a 2-butanol/methanol mixture (5:1), and PrPres was precipitated by centrifugation at 20 000 × * g* for 10 min at 20 °C. Pellets were resuspended in Laemmli sample buffer and subjected to WB. Samples were electrophoresed on NuPAGE Novex 12% Bis–Tris gels with NuPAGE MOPS-SDS running buffer in accordance with the manufacturer’s instructions (Life Technologies, Carlsbad, CA, USA). Proteins were then transferred onto Immobilon-P membranes (Millipore, Billerica, MA, USA). The blotted membranes were incubated with the anti-PrP mAbs listed in Table 1 at 4 °C overnight. After washing with PBS containing 0.05% (v/v) Tween 20, the membranes were incubated with goat anti-mouse IgG-HRP (Jackson ImmunoResearch, West Grove, PA, USA) for 60 min. Signals were developed with a chemiluminescent substrate (SuperSignal; Thermo Fisher Scientific K.K). For semi-quantification, blots were imaged using a Fluorchem system (Alpha Innotech, San Leandro, CA, USA) and analyzed using image reader software (AlphaEaseFC; Alpha Innotech) according to the manufacturer’s instructions.

### Statistical analysis

To determine statistical significance the Student’s *t*-tests were applied on paired data. Differences with *P* < 0.05 were considered significant. Statistical analysis was performed using KaleidaGraph software (Synergy Software, Reading, PA, USA).

## Results

### Characteristics of the CH1641-like scrapie isolate (Sh294) in TgOvPrP59 mice

**Figure 1:**
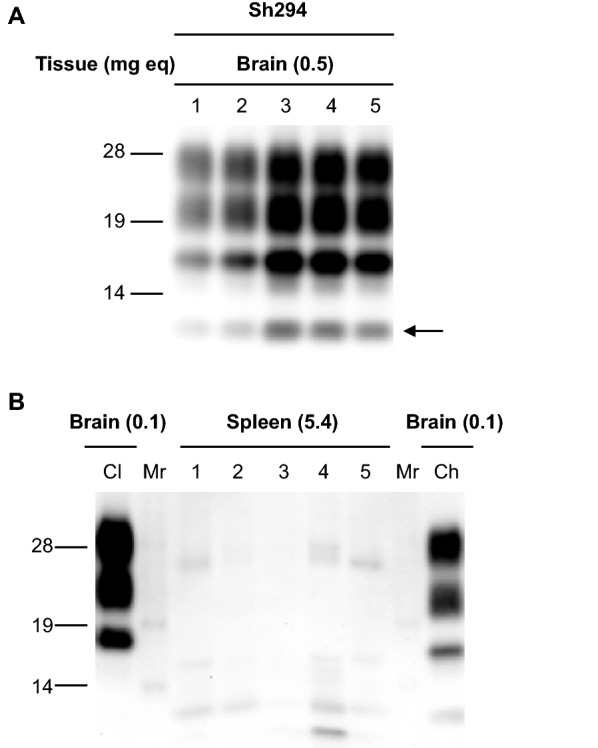
**Western blot analysis of TgOvPrP59 mice infected with Sh294.** Brains and spleens were dissected from Sh294-infected TgOvPrP59 mice at the second passage. CH1641-like PrPres banding patterns were detectable in the brains of five individual mice (lanes 1–5 in **A**), but not in the spleens of these mice (lanes 1–5 of **B**). The arrow shows the ~12-kDa C-terminal small fragment that was specific to CH1641 and CH1641-like scrapie isolates. Brain homogenates from TgOvPrP59 mice infected with classical (Cl) and CH1641-like (Sh294: Ch) scrapie isolates were loaded, for comparison of the molecular mass of unglycosylated PrPres **B**. Mr: molecular mass marker. Tissues subjected to the analysis and the equivalent tissue quantities loaded per lane are indicated on top of each panel.

First, we investigated the characteristics of Sh294 in TgOvPrP59 mice. As shown in Figure 1A, CH1641-like scrapie-specific PrPres banding patterns with the typical ~12-kDa small C-terminal fragment was detected in the brains of TgOvPrP59 mice, which were inoculated with Sh294 by WB analysis using the mAb SAF84. Splenic PrPres was detected in five out of seven TgOvPrP59 mice inoculated with brain homogenates prepared from a sheep showing the classical scrapie PrPres banding pattern (Additional file 1). In contrast, splenic PrPres was not detected in TgOvPrP59 mice infected with Sh294 at any passage number (Figure 1B and Table 2). The mean survival period of mice inoculated with Sh294 was 259 ± 24.5 at the first passage. Subsequent transmission to TgOvPrP59 mice resulted in a shortened mean survival period (215 ± 17.9 days at the third passage, Table 2).

**Table 2:** **Transmission of Sh294 to TgOvPrP59 mice**

Source	Recipient	Passage #	Survival period^a^	PrPres^b^	
				Brain	Spleen
Sh294 brain (CH1641-like scrapie)	TgOvPrP59	1	259 ± 24.5	10/10	0/10
		2	233 ± 13.4	5/5	0/5
		3	215 ± 17.9	5/5	0/5

^a^Survival periods are shown as mean survival days ± standard deviation.

^b^The number of analyzed mice is shown together with the results of PrPres detection in the brain and spleen (number of PrPres-positive mice/number of inoculated mice). All mice showed clinical signs of disease, such as clasping.

### Biochemical characteristics of PrPres from the CH1641-like scrapie isolate in TgBoPrP mice

**Figure 2:**
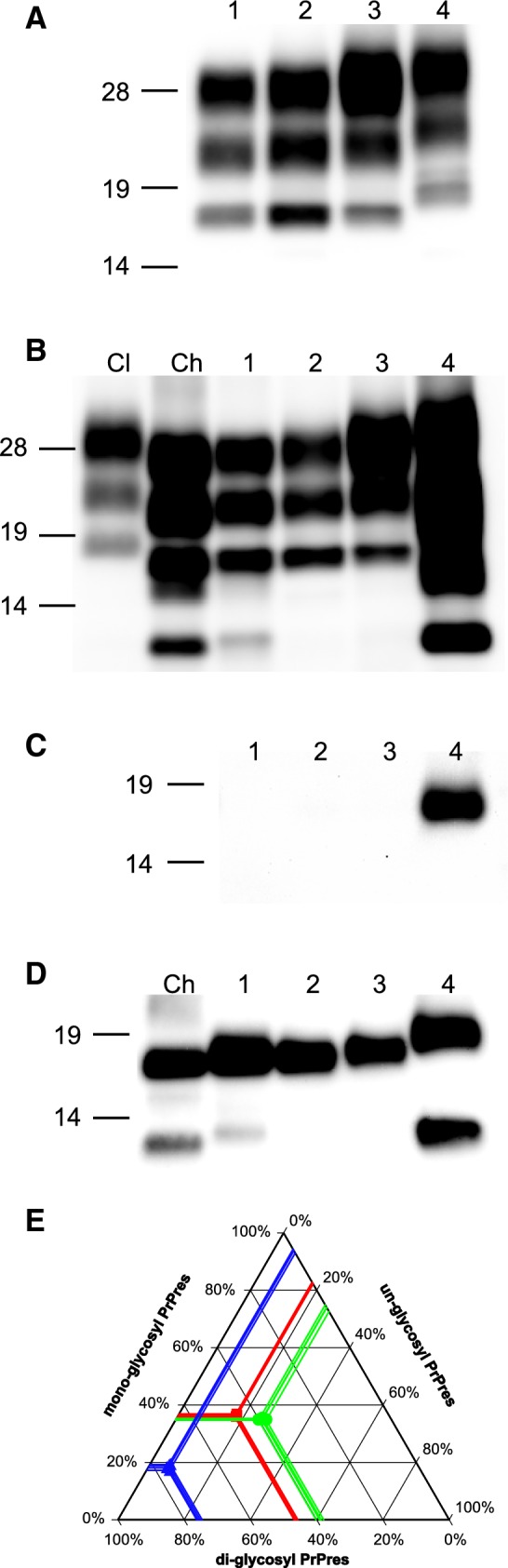
**Biochemical analysis of PrPres from Sh294 in TgBoPrP mice.** PrPres banding patterns were compared in TgBoPrP mice infected with Sh294 and BSEs using the mAbs 6H4 **A** and SAF84 **B**. PrPres was also characterized using the mAbs P4 **C** and SAF84 **D** after PNGase deglycosylation. Lane 1, Sh294; lane 2, L-BSE; lane 3, C-BSE; lane 4, H-BSE. Brain homogenates from sheep affected with classical scrapie isolate (lane Cl in **B**) and CH1641-like scrapie isolate (Sh294: lane Ch in **B**, **D**) were also loaded for comparison. Lanes from 1 to 4 in **A**, **B** were loaded with 0.6, 0.06, 0.5, and 0.4 mg brain equivalent, respectively. Lanes Cl and Ch in **B** were loaded with 0.3 and 2.0 mg brain equivalent, respectively. Lanes from 1 to 4 in **C** were loaded with 0.03, 0.006, 0.02, and 0.02 mg brain equivalent, respectively. Lanes from left to right in **D** were loaded with 0.2, 0.2, 0.06, 0.05 and 0.02 mg brain equivalent, respectively. Molecular markers are shown on the left of each panel. Glycoform profiles of PrPres in brains of TgBoPrP mice infected with Sh294, C-BSE, and L-BSE are given **E**. PrPres was detected with mAb 6H4. Symbols: red squares, Sh294; blue triangles, C-BSE; green circles, L-BSE.

PrPres banding patterns were compared between TgBoPrP mice inoculated with the Sh294 isolate and three different BSE strains. The molecular mass of PrPres in the brains of TgBoPrP mice infected with Sh294 was lower than that of TgBoPrP mice infected with H-BSE (Figure 2A). WB analysis using mAb 6H4 demonstrated identical triplet band patterns of PrPres between TgBoPrP mice infected with Sh294 and those infected with L-BSE (Figure 2A). The mAb SAF84 was used to detect the CH1641-specific ~12-kDa small C-terminal PrPres fragment in sheep affected with the Sh294 scrapie isolate (Figures 2B and D). As previously reported, this antibody also reacted with a small C-terminal fragment of PrPres in TgBoPrP mice infected with H-BSE (Figures 2C and D) [25, 26]. A similar small PrPres fragment was detected in TgBoPrP mice infected with Sh294, but not in those infected with L-BSE (Figures 2B and D). The mAb P4 reacted only with the PrPres of TgBoPrP mice infected with H-BSE. However, this antibody did not recognize the ~12-kDa small fragment (Figure 2C). Deglycosylation demonstrated that the molecular mass of the PrPres in TgBoPrP mice infected with Sh294 was slightly lower than that of TgBoPrP mice infected with C-BSE (lane 1 and 3 in Figure 2D). In contrast, TgBoPrP mice infected with Sh294 showed nearly the same molecular mass of the PrPres as L-BSE-infected TgBoPrP mice (lanes 1 and 2 in Figure 2D). WB analysis using mAb SAF84 after deglycosylation also revealed that the molecular mass of the small PrPres fragment in TgBoPrP mice infected with Sh294 was slightly higher than that in sheep Sh294 (lanes Ch and 1 in Figure 2D). Based on the signal intensities measured by the Fluorchem system, the proportions of the ~12-kDa PrPres fragment in TgBoPrP mice infected with Sh294 (~7% of total PrPres) were lower than those in sheep Sh294 (~16% of total PrPres). As shown in Figure 2E, glycoform profiles of PrPres using the mAb 6H4 were significantly different between TgBoPrP mice infected with C-BSE and Sh294 (*P *< 0.0001, Student’s *t*-test). However, similar profiles were observed in TgBoPrP mice infected with Sh294 and L-BSE. The results for transmission of the Sh294 isolate to TgBoPrP mice are given in Table 3. Although TgBoPrP mice infected with Sh294 or L-BSE exhibited unique clinical signs, including circling behavior, the survival period of L-BSE-infected TgBoPrP mice was significantly shorter than that of TgBoPrP mice infected with Sh294, even after the third passage (144 ± 4.8 days versus 172 ± 4.5 days, *P *< 0.0001, Student’s *t*-test).

**Table 3:** **Transmission of the CH1641-like scrapie isolate (Sh294) to TgBoPrP mice**

Source	Recipient	Passage #	Survival period^a^	n/n_o_^b^
Sh294 brain (CH1641-like Scrapie)	TgBoPrP	3	172 ± 4.5*	11/11
Cattle L-BSE brain	TgBoPrP	3	144 ± 4.8*	12/12
Cattle C-BSE brain	TgBoPrP	3	195 ± 4.6	14/14
Cattle H-BSE brain	TgBoPrP	3	220 ± 4.3	11/11

^a^Survival periods are shown as mean survival days ± standard deviation.

^b^Number of PrPres-positive mice out of the total number of challenged mice. All inoculated mice showed unique clinical signs of the disease (Sh294 and L-BSE, circling behavior; C-BSE, head twitching; H-BSE, constant chewing of the bedding).

* *P *< 0.0001.

### Neuropathological comparison between L-BSE and the CH1641-like scrapie isolate in TgBoPrP mice

**Figure 3:**
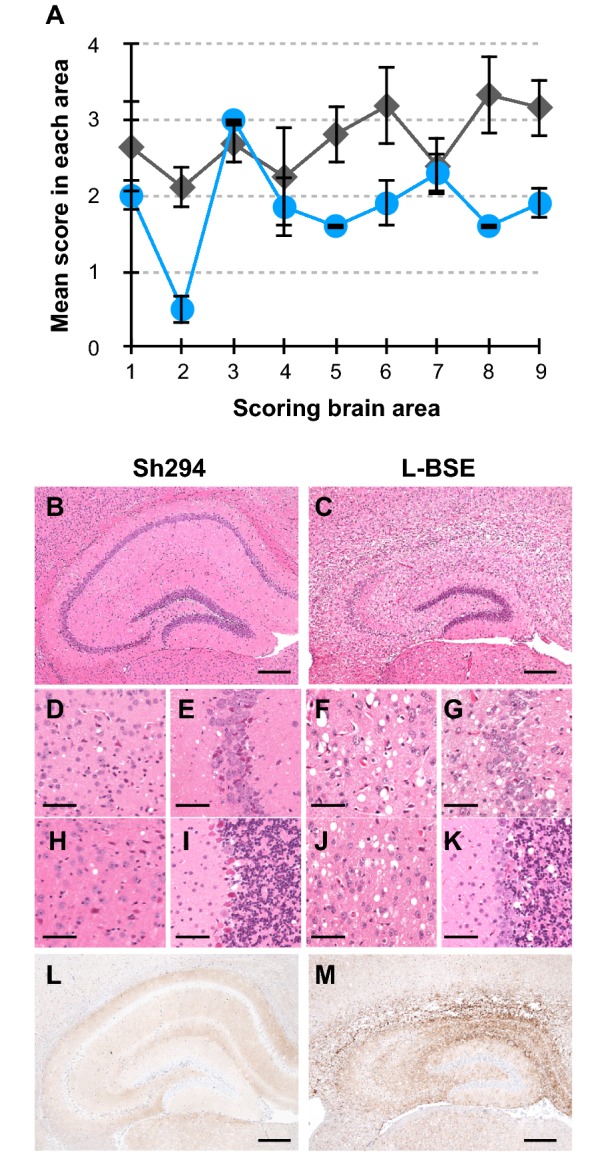
**Neuroanatomical changes and PrP**^**d**^ **accumulation in the brains of TgBoPrP mice infected with Sh294 and L-BSE.** Vacuolation in the following brain regions was scored on a scale of 0–5 **A**: 1, dorsal medulla; 2, cerebellar cortex; 3, superior cortex; 4, hypothalamus; 5, thalamus; 6, hippocampus; 7, septal nuclei of the paraterminal body; 8, cerebral cortex at the level of the hypothalamus and thalamus; 9, cerebral cortex ash the level of the septal nuclei of the paraterminal body. Results are shown as the mean ± standard deviation (circles, Sh294; diamonds, L-BSE). Sections were subjected to H&E staining. Representative examples of vacuolation in the hippocampus **B**, **C**, **E**, **G**, cortex **D**, **F**, thalamus **H**, **J**, and cerebellum **I**, **K** are shown. Representative PrP^d^ immunohistochemistry in the hippocampus is shown **L**, **M**. Scale bars indicate 200 µm **B**, **C**, **L**, **M** and 50 µm **D**–**K**.

Histological changes and PrP^d^ distributions in the brain were compared between TgBoPrP mice infected with Sh294 and L-BSE (Figure 3). TgBoPrP mice infected with L-BSE exhibited more severe spongiform changes than Sh294-infected TgBoPrP mice, except for changes in the superior cortex, hypothalamus, and the septal nuclei of the paraterminal body (Figures 3A–K). Neuronal cells in the CA1 area were particularly rarefied in L-BSE-infected TgBoPrP mice as compared with those in the CA1 area of Sh294-infected mice (Figures 3B and C). Immunohistochemistry demonstrated the different PrP^d^ distribution patterns between TgBoPrP mice infected with Sh294 and L-BSE. Many granular PrP^d^ deposits were detected in the hippocampus of L-BSE-infected TgBoPrP mice but not Sh294-infected mice (Figures 3L and M).

### Interspecies transmission of the CH1641-like scrapie isolate passaged in TgBoPrP mice to wild-type mice

**Figure 4:**
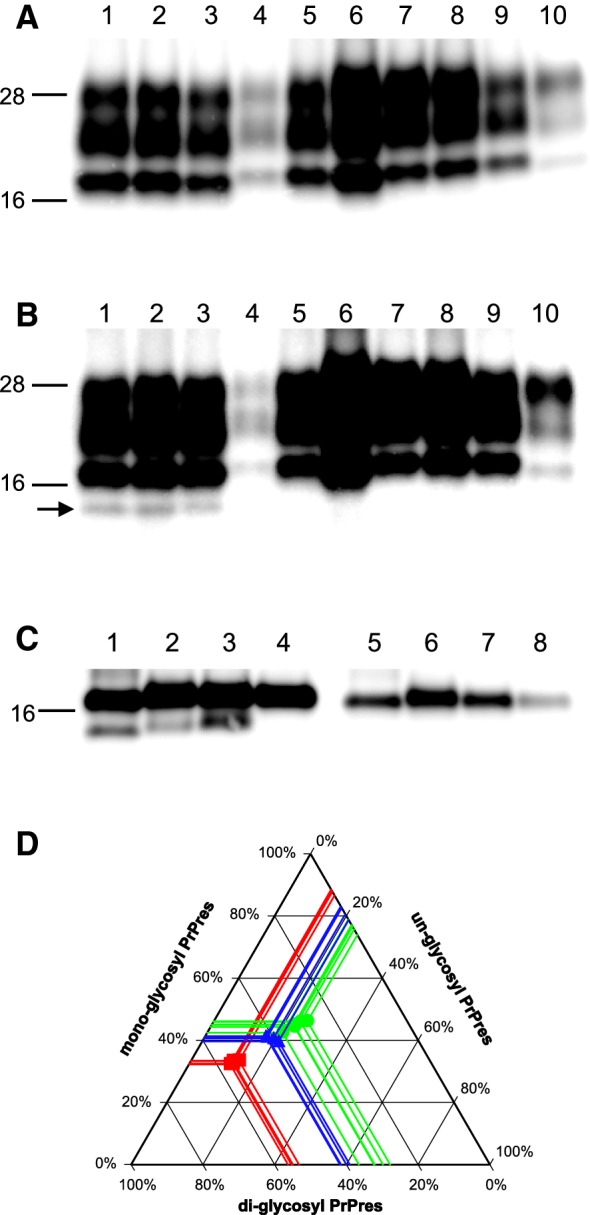
**Biochemical analysis of PrPres accumulated in the brains of ICR mice infected with TgBo-passaged Sh294.** PrPres banding patterns were compared among ICR mice infected with TgBo-passaged Sh294, sheep-passaged L-BSE, mouse-adapted laboratory classical scrapie strains, and mouse-adapted C-BSE using the mAb 6H4 **A** and SAF84 **B**. Lane 1, TgBo-passaged Sh294 (first passage in ICR); lane 2, TgBo-passaged Sh294 (second passage in ICR); lane 3, TgBo-passaged Sh294 (third passage in ICR); lane 4, sheep-passaged L-BSE (first passage in ICR); lane 5, sheep-passaged L-BSE (second passage in ICR); lane 6, sheep-passaged L-BSE (third passage in ICR); lane 7, ME7; lane 8, 22L; lane 9, Chandler; lane 10, mouse-adapted C-BSE (mBSE). Laboratory strains (ME7, 22L, Chandler, and mBSE) were serially passaged in ICR mice (more than 10 passages). Lanes 1–3, 4 and 5, 6, and 7–10 in **A**, **B** were loaded with 0.5 mg, 2 mg, 0.1 mg, and 0025 mg brain equivalent, respectively. PrPres was also characterized using the mAbs SAF84 (**C**, lane 1–4) and 6H4 (**C**, lane 5–8) after PNGase deglycosylation. 1, sheep Sh294; 2, TgBoPrP mouse infected with Sh294; 3, ICR mouse infected with TgBo-passaged Sh294; 4, TgBoPrP mouse infected with L-BSE. Lanes 1–3 and 5–7 in **C** were loaded with 0.25 mg brain equivalent. Lanes 4 and 8 in **C** were loaded with 0.003 mg brain equivalent. The arrow in **B** shows the ~12-kDa C-terminal small fragment. Molecular markers are shown on the left of each panel. Glycoform profiles of PrPres from ICR mice infected with TgBo-passaged Sh294 (third passage), sheep-passaged L-BSE (third passage) and mBSE (14^th^ passage) are given **D**. PrPres was detected with mAb 6H4. Symbols: red squares, mBSE; blue triangles, sheep-passaged L-BSE; green circles, TgBo-passaged Sh294.

**Figure 5:**
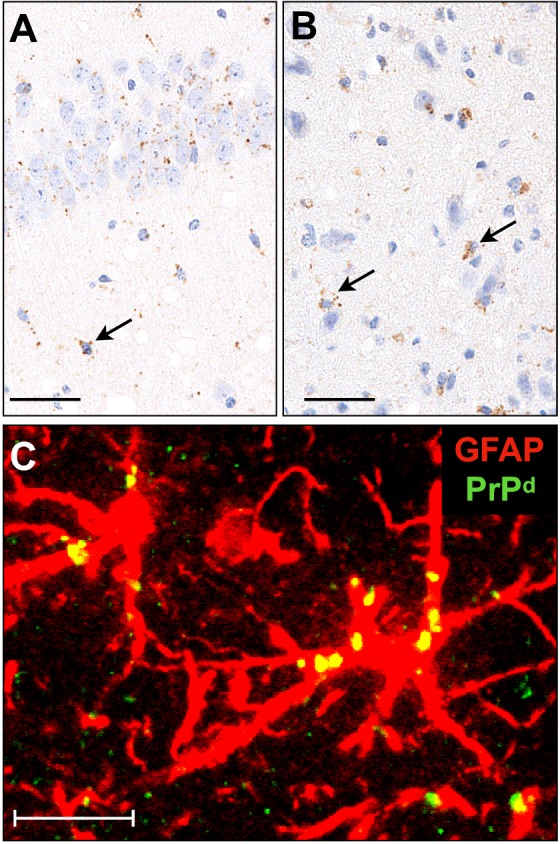
**PrP**^**d**^ **distribution in the brains of ICR mice infected with TgBo-passaged Sh294.** Sections of the hippocampus and thalamus were subjected to immunostaining for PrP^d^ **A**–**C** and GFAP **C**. Granular PrP^d^ was dispersed in neuronal cells, glial cells (arrows), and neuropils **A**, **B**. Some PrP^d^ signals were detected in the cytoplasm of hypertrophied astrocytes (yellow) **C**. Scale bars indicate 50 µm **A**, **B** and 10 µm **C**.

To improve the understanding of the differences between Sh294 and L-BSE in TgBoPrP mice, interspecies transmission of TgBo-passaged Sh294 to ICR mice was conducted. Although none of the ICR mice injected with TgBo-passaged Sh294 developed any clinical signs of the disease at the first passage, PrPres was detected by WB analysis in seven of twenty mice sacrificed at the end of their lifespan (Table 4). The mean survival period of mice exhibiting an accumulation of PrPres was 807 ± 46.9 days. Subsequent transmission to ICR mice resulted in a shortened mean survival period (515 ± 33.6 days) with a 100% infection rate. The third passage in ICR mice further reduced the mean survival period (453 ± 40.4 days). At the third passage, all inoculated mice accumulated PrP^d^ in the brain, and some of them developed clinical signs of the disease, such as rough fur and kyphosis. The sudden death of two out of six inoculated mice occurred at day 477 and 552, respectively, without any clinical signs. However, post-mortem analysis revealed that they accumulated PrP^d^ in the brain. In contrast, ICR mice infected with sheep-passaged L-BSE still showed a more prolonged survival period compared with that in mice infected with TgBo-passaged Sh294 (Table 4). The PrPres banding patterns of ICR mice infected with TgBo-passaged Sh294 was different from those of ICR mice infected with sheep-passaged L-BSE (Figures 4A and B). WB analysis using the mAb SAF84 demonstrated reproduction of CH1641-like scrapie-specific PrPres banding patterns with the ~12-kDa small fragment in ICR mice infected with TgBo-passaged Sh294 (arrow in Figure 4B and lane 3 in Figure 4C). This small fragment could not be detected with mAb 6H4 (lane 1–3 in Figure 4A and lane 7 in Figure 4C). These results indicate that the small fragment detected in ICR mice infected with TgBo-passaged Sh294 shares similar properties with the C-terminal PrPres fragment detected in sheep Sh294 and TgBoPrP mice infected with Sh294. Deglycosylation demonstrated that the molecular mass of the small C-terminal PrPres fragment was identical between TgBoPrP mice infected with Sh294 and ICR mice infected with TgBo-passaged Sh294, but slightly higher than that of the original sheep Sh294 (lane 1–3 in Figure 4C). Glycoform profiles using the mAb 6H4 demonstrated that the mono-glycosylated PrPres was dominant in the brain of ICR mice infected with TgBo-passaged Sh294 as well as in the brain of ICR mice infected with sheep-passaged L-BSE (Figure 4D). In contrast, the amount of diglycosylated PrPres was dominant in ICR mice infected with mBSE (Figure 4D). Granular PrP^d^ deposits were detected in neuronal cells and astrocytes of the hippocampus and thalamus of ICR mice infected with TgBo-passaged Sh294 (Figures 5A and B). Some PrP^d^ signals were detected in the cytoplasm of hypertrophied astrocytes (Figure 5C).

**Table 4:** **Transmission of TgBo-passaged Sh294 to wild-type mice**

Source	Recipient	Passage #	Survival period^a^	n/n_o_^b^
TgBoPrP mouse brain infected with Sh294	ICR	1	807 ± 46.9	7/20
		2	515 ± 33.6*	6/6^c^
		3	453 ± 40.4*	8/8^d^
Sheep brain infected with L-BSE	ICR^f^	1	710^d^	1/15^e^
		2	680 ± 20.2	7/9
		3	702 ± 29.5	9/11

^a^Survival periods are shown as mean survival days ± standard deviation.

^b^Number of PrPres-positive mice out of the total number of challenged mice.

^c^Two out of six mice were suddenly dead without any clinical signs at 477^th^ and 552^th^ day. The other four mice developed clinical signs of the disease, such as emaciation, rough fur and kyphosis.

^d^Two out of eight inoculated mice were suddenly dead without any clinical signs at 377^th^ and 441^th^ day, respectively. The other six mice developed the same clinical signs as above.

^e^The data for the 1^st^ passage obtained from our previous study shown in the reference (20).

^f^ICR mice infected sheep-passaged L-BSE did not show any clinical even at the 3^rd^ passage.

* *P *< 0.05.

## Discussion

The biological and biochemical properties of the experimental CH1641-like scrapie isolate (Sh294) in TgBoPrP mice were different from those of H- and C-BSE [25]. In contrast, TgBoPrP mice infected with Sh294 exhibited clinical signs and the triplet band pattern of PrPres similar to those of mice infected with L-BSE. However, the ~12-kDa small C-terminal fragment of PrPres could only be detected in TgBoPrP mice infected with Sh294. Moreover, the mean survival periods, lesion profiles, and PrP^d^ distribution patterns in the brain were distinctly different between the mice infected with Sh294 and those infected with L-BSE. Interspecies transmission of TgBo-passaged Sh294 to wild-type (ICR) mice also demonstrated the biological differences between Sh294 and L-BSE passaged in TgBoPrP mice. It is known that L-BSE from cattle or TgBoPrP mice is unable to transmit to ICR mice [20, 27]. In contrast, TgBo-passaged Sh294 can transmit to ICR mice even though inefficiently. Since we previously reported that L-BSE could transmit to ICR mice after cross-species transmission to sheep [20], we can now compare the characteristics of the CH1641-like scrapie isolate and L-BSE in wild-type mice. The mean survival period of ICR mice infected with sheep-passaged L-BSE was significantly extended in comparison with that of ICR mice infected with TgBo-passaged Sh294 (Table 4). Although the glycoform profiles were similar between ICR mice infected with TgBo-passaged Sh294 and those infected with sheep-passaged L-BSE, the molecular mass of PrPres was distinctly different between them. In the histopathological analysis, florid plaques were detected in the brains of ICR mice infected with sheep-passaged L-BSE [20], but not in the brains of mice infected with TgBo-passaged Sh294. Thus, all our data demonstrate that the Sh294 isolate is independent of all three BSE strains, suggesting that CH1641-like scrapie isolates could not be candidates for the origin of BSEs. Indeed, several studies have suggested that spontaneously occurring atypical BSEs in cattle may have been the origin of C-BSE [28–32].

It is of interest to us that TgBo-passaged Sh294 is transmitted to ICR mice with the CH1641-like scrapie-specific PrPres banding patterns because this scrapie isolate cannot transmit directly from sheep to wild-type mice [19]. The biological and biochemical features of TgBo-passaged Sh294 in ICR mice were evidently different from those of sheep-passaged L-BSE. Moreover, these features were also different from all other tested laboratory scrapie strains (22L, ME7, and Chandler), mouse-adapted Japanese scrapie isolates, and the mBSE strain, which were available in our laboratory (Figures 4A, B, D, and Additional file 2) [33, 34]. To the best of our knowledge, only a group reported the transmission of French natural CH1641-like scrapie isolates from sheep to wild-type (C57BL/6) mice [35]. In contrast to our results, these isolates easily transmitted to C57BL/6 mice. However, the PrPres banding patterns and the histopathological properties observed in diseased mice exhibited a classical scrapie phenotype. This discrepancy could be explained by the co-existence of two different scrapie prions in French natural CH1641-like scrapie isolates, i.e., a classical scrapie prion and a CH1641 scrapie prion. This notion could be supported by the previous finding that some of TgOvPrP4 mice, overexpressing ovine PrP_ARQ_, exhibited the classical scrapie PrPres banding patterns after inoculation with the French natural CH1641-like scrapie isolates [36, 37]. It has also been reported that TgOvPrP4 mice inoculated with French natural CH1641-like scrapie isolates accumulate PrPres in both the brain and spleen [38]. Although TgOvPrP4 and TgOvPrP59 transgenic mouse lines were derived from different founders, they overexpressed approximately three times as much PrP_ARQ_ in the brain under the control of the neuron-specific enolase promoter as that in sheep brains [21]. Since the previous report failed to detect the expression of ovine PrP mRNA in non-nervous tissues of TgOvPrP4 mice by RT-PCR [39], the precise mechanisms by which PrP^d^ accumulates in the spleen of this transgenic mouse line infected with classical scrapie isolates remain unknown. However, considering the ovine PrP^C^ expression level and similar response to the classical scrapie isolate, TgOvPrP59 mice may be a useful model to examine the similarity of the Sh294 isolate to French natural CH1641-like isolates. We demonstrated that none of the Sh294 inoculated mice exhibited the PrPres banding patterns of classical scrapie, even after the third passage in TgOvPrP59 mice. Moreover, splenic PrPres was detected in five out of seven TgOvPrP59 mice inoculated with the classical scrapie isolate, but not in any TgOvPrP59 mice inoculated with Sh294 (Additional file 1 and Table 2). Likewise, TgOvPrP4 mice inoculated with the British CH1641 scrapie isolate have been reported not to accumulate PrPres in their spleens [38]. These data strongly support that the experimental Sh294 isolate consisted of a single scrapie prion, similar to the CH1641 scrapie prion found in the UK. Unlike natural CH1641-like scrapie isolates, selection of a particular scrapie prion might occur through the experimental intraspecies transmission in sheep. Further transmission study of the British CH1641 scrapie isolate to TgOvPrP59 mice is needed to elucidate the similarity between the British CH1641 scrapie isolate and the Sh294 isolate.

The CH1641 scrapie prion remains poorly understood. In this study, we first demonstrated that the CH1641-like scrapie isolate was independent of BSEs, including atypical BSE. Then, we showed that the range of host species susceptible to the CH1641-like scrapie isolate changed via an interspecies transmission route. Finally, we demonstrated the successful transmission of the CH1641-like scrapie isolate (Sh294) to wild-type mice with its specific PrPres banding patterns. Our findings improve the current understanding of the relationship between CH1641-like scrapie isolates and BSEs, including atypical BSE.

## Additional files

**Additional file 1.** **PrPres accumulation in the spleen of TgOvPrP59 mice infected with a classical scrapie isolate.** Brains and spleens were dissected from TgOvPrP59 mice inoculated with brain homogenates prepared from a sheep showing the classical scrapie PrPres banding patterns. PrPres was detected with the mAb SAF84. Five out of seven mice (lanes 1–7 of the panels) accumulated the PrPres in the spleen. Brain homogenates from TgOvPrP59 mice infected with classical (Cl) and CH1641-like (Ch) scrapie isolates were loaded for comparison of the molecular mass of unglycosylated PrPres. Tissues subjected to the analysis and the equivalent tissue quantities loaded per lane are indicated on top of the panel (B).

**Additional file 2.** **Lesion profiles of ICR mice infected with TgBo-passaged Sh294.** Vacuolation in the brain regions was scored (A). The brain regions are indicated in Figure 1. Results are shown as the mean ± standard deviation (circles, TgBo-passaged Sh294 at the third passage; squares, 22L; diamonds, Chandler).

## Abbreviations

BSE: bovine spongiform encephalopathy
PrP: prion protein
PrP^d^: disease-associated PrP
PrPres: protease-resistant core of PrP^d^
PrP^C^: cellular PrP
TSE: transmissible spongiform encephalopathy
WB: Western blot
TgBoPrP mice: bovine PrP overexpressing mice
TgOvPrP59: ovine PrP_ARQ_ overexpressing mice
PrP_ARQ_: PrP showing the triplet sequences at codons 136, 154 and 171
mBSE: mouse-adapted classical BSE
GFAP: glial fibrillary acidic protein
H&E: hematoxylin and eosin
mAb: monoclonal antibody
