# Supplementary figures and images for: Interspecies transmission to bovinized transgenic mice uncovers new features of a CH1641-like scrapie isolate
Source: Vet Res. 2018 Nov 28;49:116. doi: 10.1186/s13567-018-0611-1 (PMC6262972; doi:10.1186/s13567-018-0611-1)

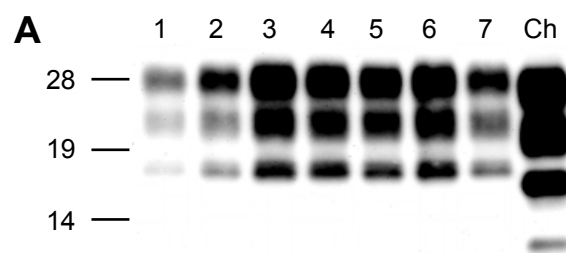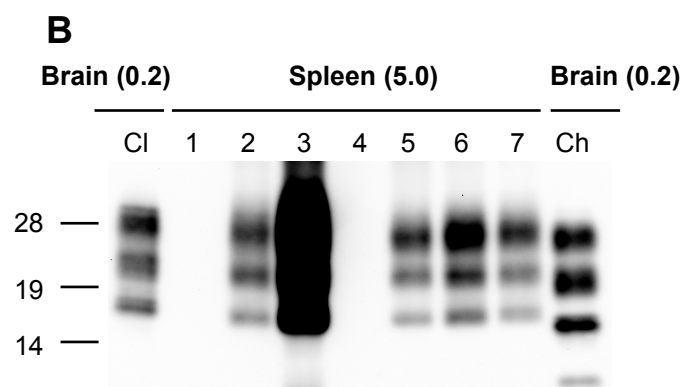

Supplement: Supplementary file 1 — Additional file 1. PrPres accumulation in the spleen of TgOvPrP59 mice infected with a classical scrapie isolate. Brains and spleens were dissected from TgOvPrP59 mice inoculated with brain homogenates prepared from a sheep showing the classical scrapie PrPres banding patterns. PrPres was detected with the mAb SAF84. Five out of seven mice (lanes 1–7 of the panels) accumulated the PrPres in the spleen. Brain homogenates from TgOvPrP59 mice infected with classical (Cl) and CH1641-like (Ch) scrapie isolates were loaded for comparison of the molecular mass of unglycosylated PrPres. Tissues subjected to the analysis and the equivalent tissue quantities loaded per lane are indicated on top of the panel (B). [file 13567_2018_611_MOESM1_ESM.pdf]

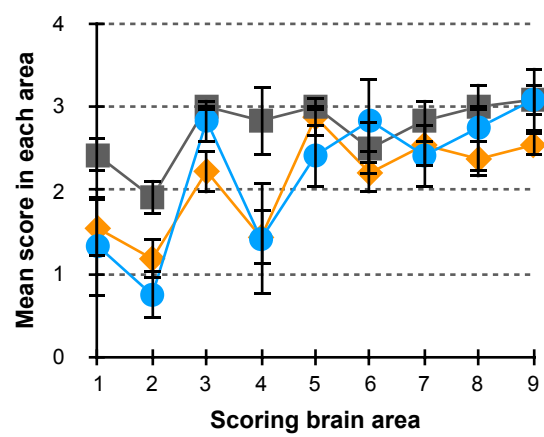

Supplement: Supplementary file 2 — Additional file 2. Lesion profiles of ICR mice infected with TgBo-passaged Sh294. Vacuolation in the brain regions was scored (A). The brain regions are indicated in Figure 1. Results are shown as the mean ± standard deviation (circles, TgBo-passaged Sh294 at the third passage; squares, 22L; diamonds, Chandler). [file 13567_2018_611_MOESM2_ESM.pdf]
